# Supplementary material for: An In Vitro Cell Culture Model for Pyoverdine-Mediated Virulence
Source: Pathogens. 2020 Dec 24;10(1):9. doi: 10.3390/pathogens10010009 (PMC7824568; doi:10.3390/pathogens10010009)
Supplement: Supplementary file 1 [file pathogens-10-00009-s001.pdf]

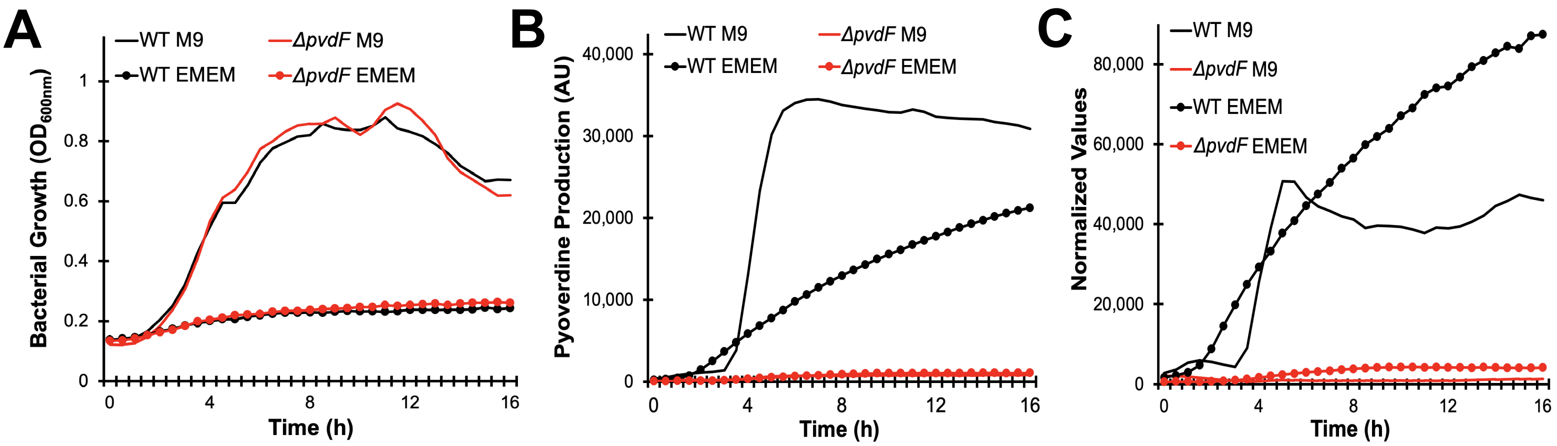

Figure S1. ***P. aeruginosa*** **produces pyoverdine in serum-free cell culture medium.** Bacterial growth **(A)**, pyoverdine production **(B)**, or pyoverdine production normalized to growth **(C)** by wild-type PAO1 or PAO1*ΔpvdF* in M9 medium or Eagle's Minimal Essential Medium (EMEM).

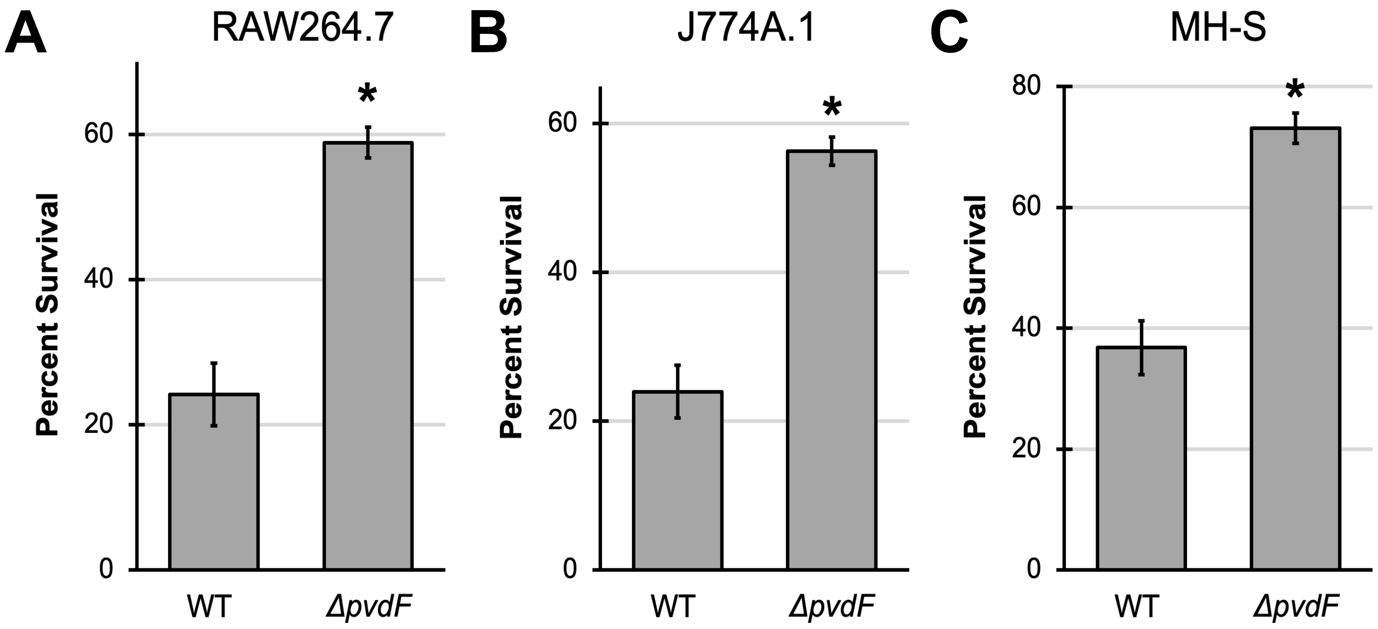

Figure S2. Pyoverdine production is important for virulence in different murine macrophage cell lines. Viability of RAW264.7 **(A)**, J774A.1 **(B)**, or MH-S **(C)** murine macrophages after 1.5 h exposure to filtrates from wild-type PAO1 or PAO1*ΔpvdF*. Error bars represent SEM from four biological replicates. * corresponds to $p < 0.01$, as determined by Student's *t*-test.

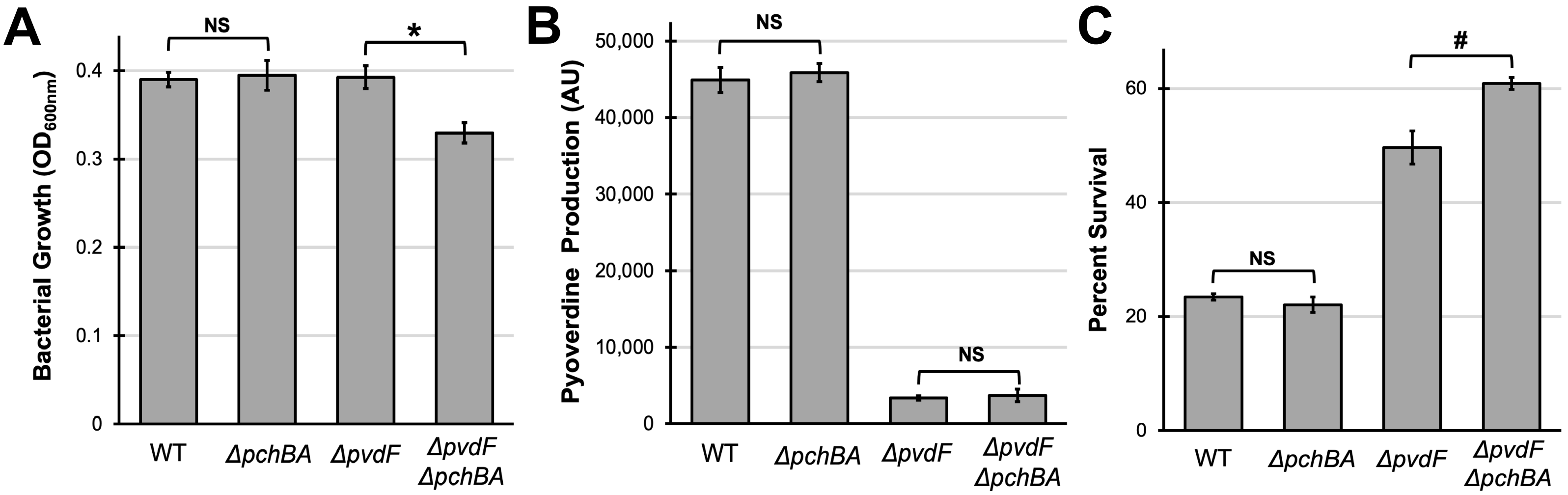

Figure S3. ***P. aeruginosa*** **filtrate toxicity is independent of pyochelin production. (A, B)** Bacterial growth **(A)** and pyoverdine production **(B)** of wild-type *P. aeruginosa* PAO1, pyochelin biosynthetic mutant PAO1*ΔpchBA*, pyoverdine biosynthetic mutant PAO1*ΔpvdF*, or the pyochelin, pyoverdine double mutant PAO1*ΔpvdFΔpchBA* after 16 h incubation in EMEM. **(C)** Murine macrophage viability after 1.5 h exposure to bacterial filtrates. Error bars represent SEM from four biological replicates. * corresponds to $p < 0.01$, # corresponds to $p < 0.05$, and NS corresponds to $p > 0.05$, as determined by Student's *t*-test.

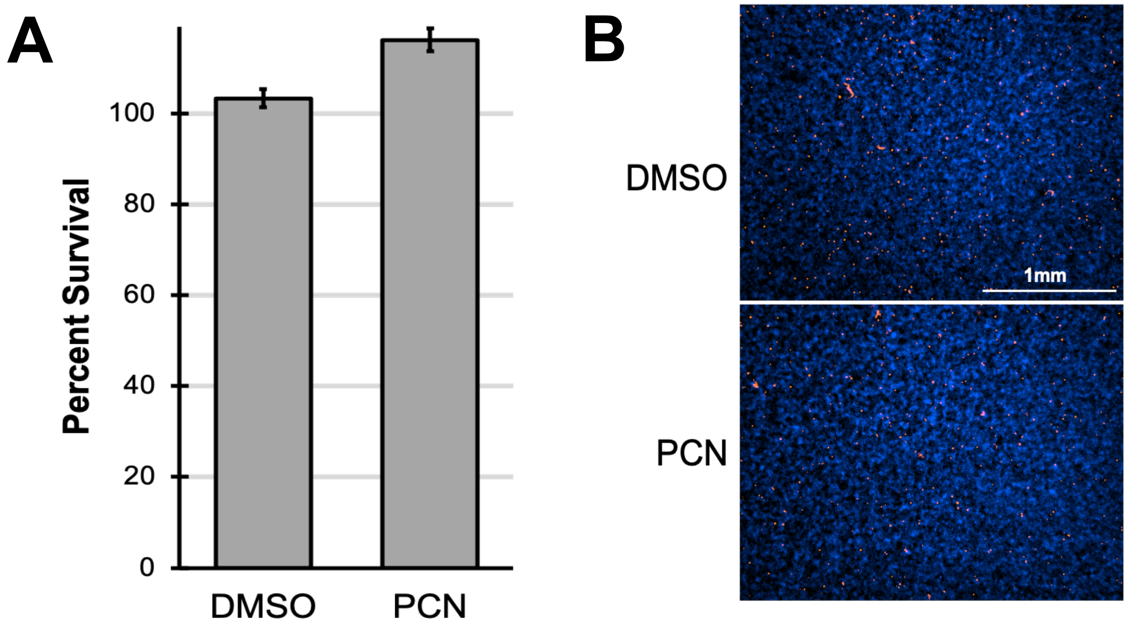

Figure S4. Pyocyanin does not contribute to filtrate toxicity. (A) Murine macrophage viability after 1.5 h exposure to EMEM supplemented with 5 μM pyocyanin (PCN) or DMSO solvent control. Cell viability was measured using an alamarBlue assay. Error bars represent SEM from three biological replicates. **(B)** Macrophages labeled with Hoechst 33342 (blue) or Sytox Orange (red) following 1.5 h exposure to EMEM supplemented with 5 μM PCN or solvent control.

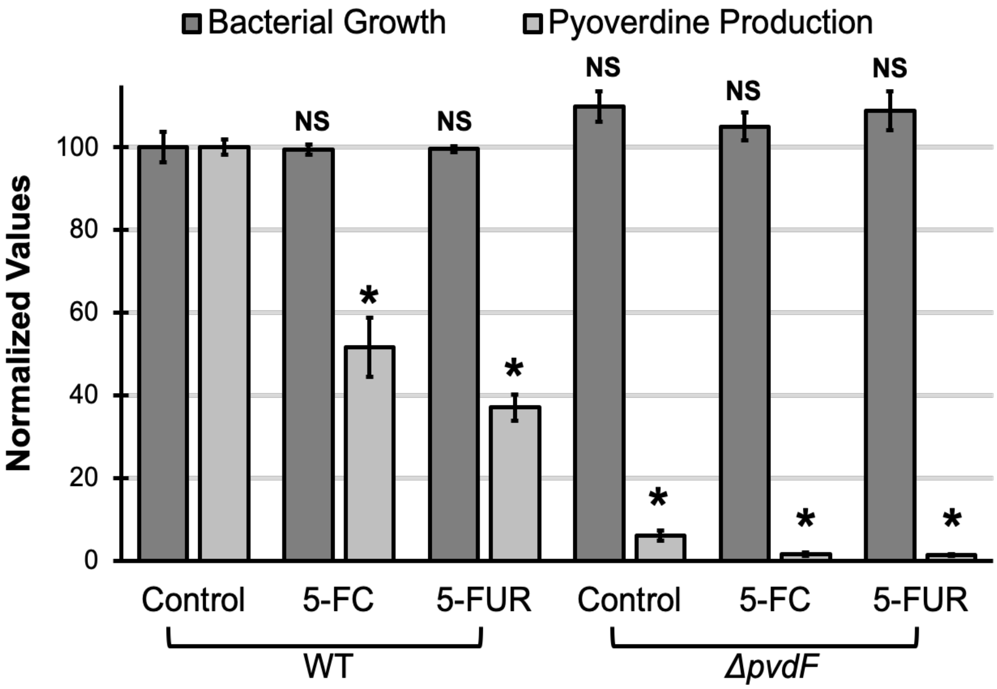

Figure S5. Fluoropyrimidines function as pyoverdine antivirulents. Relative bacterial growth and pyoverdine production of wild-type PAO1 or PAO1*ΔpvdF* in the presence of 50 μM 5-fluorocytosine (5-FC) or 10 μM 5-fluorouridine (5-FUR) in EMEM. Error bars represent SEM from three biological replicates. * corresponds to $p < 0.01$ and NS corresponds to $p > 0.05$, as determined by Student's t-test.

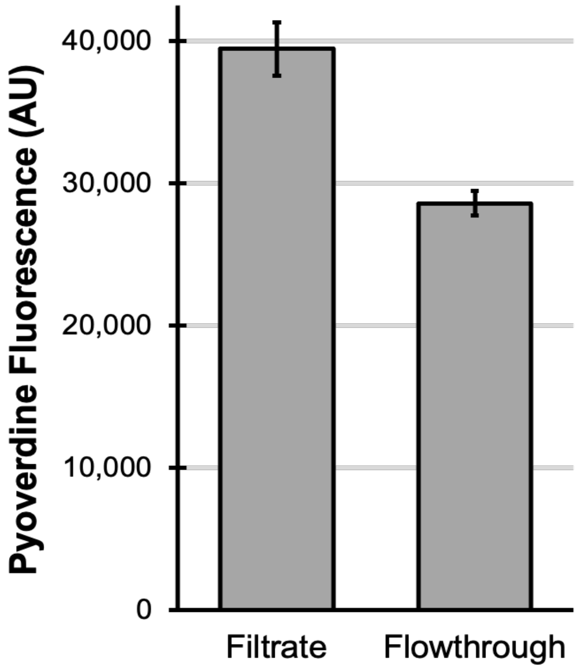

Figure S6. Pyoverdine is present in the low-molecular-weight fraction of the filtrate (flowthrough). Pyoverdine fluorescence in wild-type *P. aeruginosa* PAO1 filtrate before and after centrifugal filtration to remove molecules heavier than 5 kDa. Error bars represent SEM from six biological replicates.

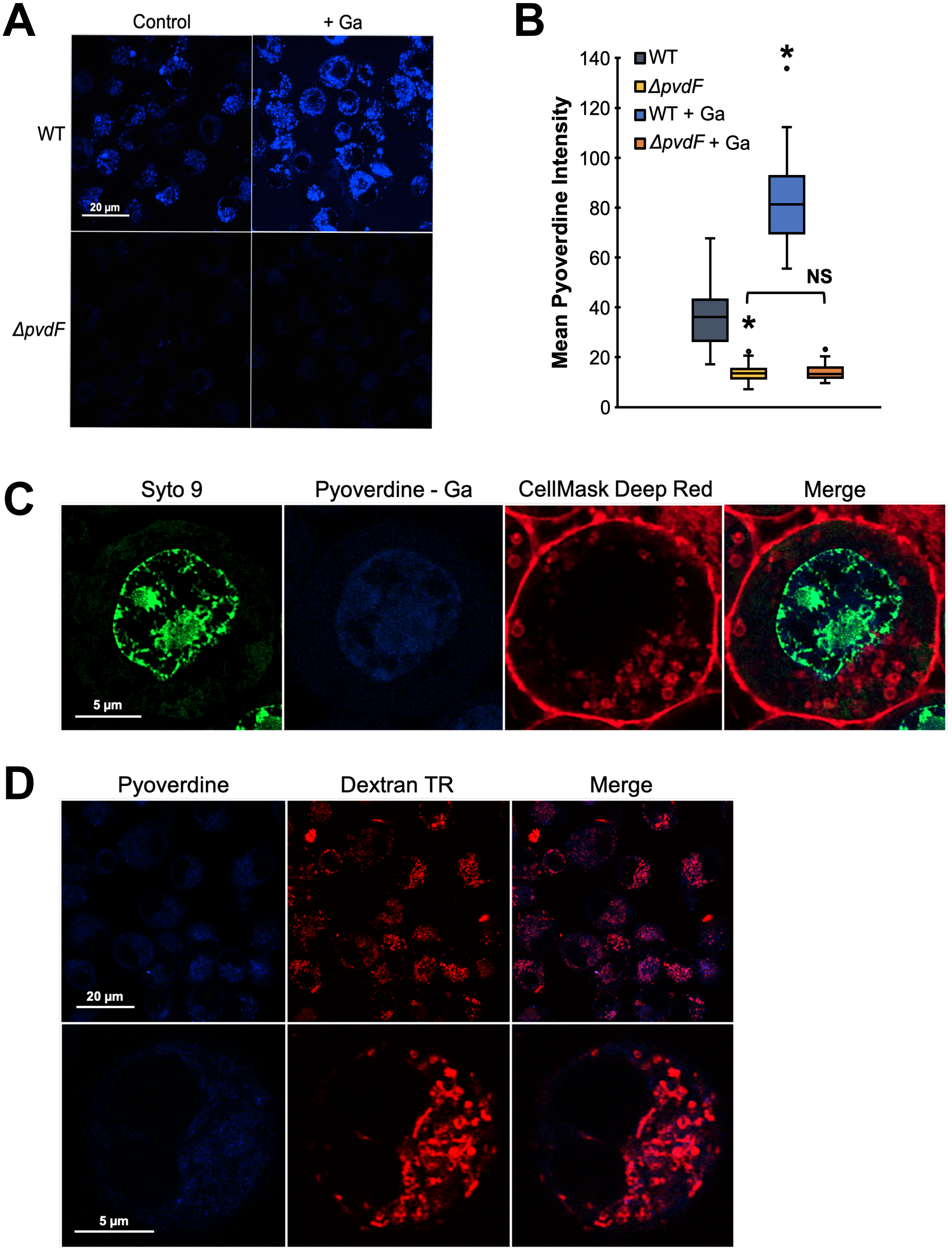

Figure S7. Intracellular pyoverdine fluorescence is not observed in cells treated with pyoverdine-deficient material. (A) Confocal laser-scanning microscopy of macrophages exposed to flowthrough materials containing pyoverdine or gallium (III)-complexed pyoverdine for 1.5 h. Flowthrough was pretreated with gallium (III) nitrate or solvent control. **(B)** Quantification of pyoverdine fluorescence within 50 individual cells. **(C)** Intracellular fluorescence of gallium (III)-complexed pyoverdine in cells treated with PAO1*ΔpvdF* flowthrough supplemented with gallium (III) nitrate. Cells were counterstained with Syto9 nucleic acid stain (green) and CellMask plasma membrane stain (red). **(D)** Macrophages treated with PAO1*ΔpvdF* flowthrough and 10,000 MWCO dextran labeled with Texas Red for 1.5 h. The bottom row shows enlarged micrographs of one representative cell. * corresponds to $p < 0.01$ and NS corresponds to $p > 0.05$, as determined by Student's *t*-test.
